# Comprehensive Proteomic Profiling of Pressure Ulcers in Patients with Spinal Cord Injury Identifies a Specific Protein Pattern of Pathology

**DOI:** 10.1089/wound.2019.0968

**Published:** 2020-03-19

**Authors:** Montserrat Baldan-Martin, Tatiana Martin-Rojas, Nerea Corbacho-Alonso, Juan Antonio Lopez, Tamara Sastre-Oliva, Felix Gil-Dones, Jesus Vazquez, Jose Manuel Arevalo, Laura Mourino-Alvarez, Maria G. Barderas

**Affiliations:** ^1^ Department of Vascular Physiopathology, National Hospital for Paraplegics (HNP), SESCAM, Toledo, Spain.; ^2^ Cardiovascular Proteomics Laboratory and CIBER-CV, CNIC, Madrid, Spain.; ^3^ Department of Plastic Surgery, National Hospital for Paraplegics (HNP), SESCAM, Toledo, Spain.

**Keywords:** pressure ulcer, spinal cord injury, proteomics, tandem mass tags

## Abstract

**Objective:** Severe pressure ulcers (PUs) do not respond to conservative wound therapy and need surgical repair. To better understand the pathogenesis and to advance on new therapeutic options, we focused on the proteomic analysis of PU, which offers substantial opportunities to identify significant changes in protein abundance during the course of PU formation in an unbiased manner.

**Approach:** To better define the protein pattern of this pathology, we performed a proteomic approach in which we compare severe PU tissue from spinal cord injury (SCI) patients with control tissue from the same patients.

**Results:** We found 76 proteins with difference in abundance. Of these, 10 proteins were verified as proteins that define the pathology: antithrombin-III, alpha-1-antitrypsin, kininogen-1, alpha-2-macroglobulin, fibronectin, apolipoprotein A-I, collagen alpha-1 (XII) chain, haptoglobin, apolipoprotein B-100, and complement factor B.

**Innovation:** This is the first study to analyze differential abundance protein of PU tissue from SCI patients using high-throughput protein identification and quantification by tandem mass tags followed by liquid chromatography tandem mass spectrometry.

**Conclusion:** Differential abundance proteins are mainly involved in tissue regeneration. These proteins might be considered as future therapeutic options to enhance the physiological response and permit cellular repair of damaged tissue.

**Figure:**
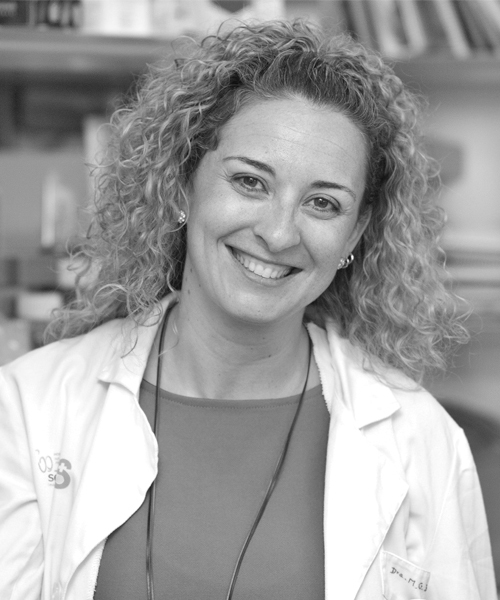
**Maria G. Barderas, PhD**

## Introduction

Spinal cord injury (SCI) is the result of trauma at any level of the spinal cord causing temporary or permanent damage, and has a significant effect on the patient's physical and psychosocial well-being.^1^ SCI is associated with considerable health care costs, morbidity, and mortality, especially when it reaches advanced stages. Despite advances in health care technology, pressure ulcers (PUs), also called pressure injuries, remain an all too common complication in patients with SCI. PU formation is a complex and poorly understood process, and its prevalence significantly increases with time postinjury.^2,3^ Indeed, recurrence of PUs after healing has been reported to be as high as 35% for patients with SCI.^4^ PUs are defined as lesions on any skin surface that result from localized shear and/or compression for a prolonged period over bony prominences at certain anatomic locations (*e.g*., sacrum).^5–7^ This occurrence leads to ischemia of overlying soft tissues that can ultimately result in necrosis.^7,8^ Often, severe PUs (grade 3 or 4 in the grading system, *i.e*., with full thickness skin loss) do not respond to conservative wound therapy, and surgery is required to prevent further tissue damage.^9^ These limitations in the therapeutic strategies used for PUs underscore the urgent need for new treatments for this serious public health problem.

Although enormous efforts have been expended to better understand the main risk factors for PUs and to improve prevention, the course of these lesions hampers an accurate and individualized evaluation. In accordance, new tools are desirable to further our knowledge on the cellular/molecular subjacent mechanisms of PU development.

**Figure 1.**
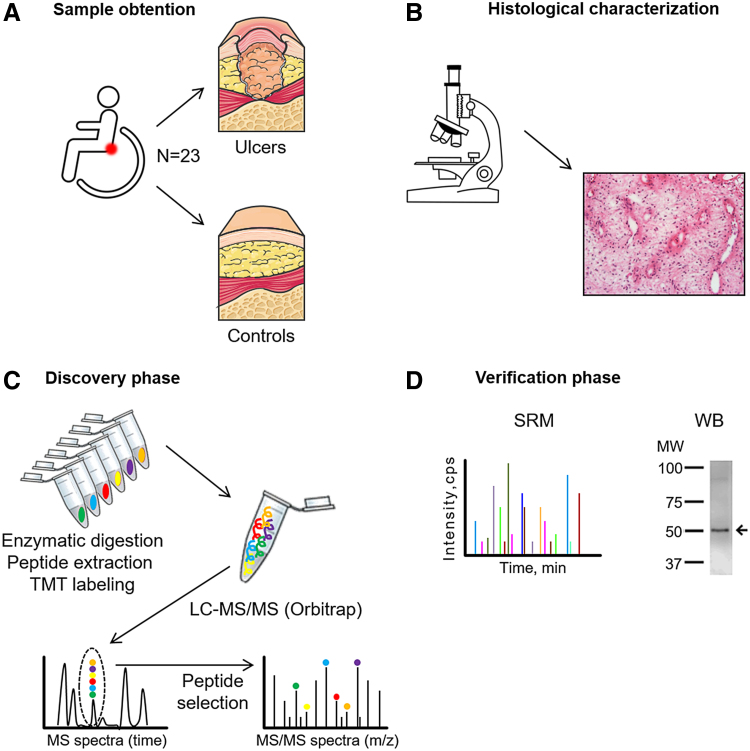
Schematic representation of the workflow. **(A)** Samples were collected from patients with spinal cord injury. Control and PU tissues were analyzed from the same patient. **(B)** Hematoxylin and eosin staining was performed to evaluate the morphological changes in the samples. **(C)** Discovery phase was performed using TMT labeling followed by LC-MS/MS. **(D)** Verification phase was performed with an independent cohort of patients and employing two orthogonal techniques, SRM and western blotting (WB). LC-MS/MS, liquid chromatography tandem mass spectrometry; PU, pressure ulcer; SRM, selected reaction monitoring; TMT, tandem mass tags. Color images are available online.

Proteomics technologies offer substantial opportunities to identify significant changes in protein abundance during the course of PU formation in an unbiased manner. Advancements in proteomics technology allow the molecular determinants of complex samples like tissue to be analyzed using mass spectrometry (MS). In this study, we describe, for the first time to our knowledge, the protein pattern that defines severe PUs (stages 3 and 4) in SCI patients categorized as grades A and B in the American Spinal Injury Association (ASIA) scale.^10^ We used tandem mass tags (TMT), a powerful and novel method for quantitative proteome analysis developed in recent years utilizing isobaric mass tags,^11–13^ to construct a comparative proteomic profile of PU and control tissue from the same patients (Fig. 1). The use of these methods can permit the identification of important biological processes that are altered in PU, and may enable the discovery of new therapeutic options to improve clinical management of these patients.

## Clinical Problem Addressed

PUs in stages 3 and 4 are a common complication in patients with SCI who do not respond to conservative wound therapy and need surgical repair.^9^ PUs are defined as a lesion on any skin surface that results from pressure or pressure in combination with shear force and/or friction.^5–7^ New tools are desirable to further our knowledge regarding the cellular/molecular subjacent mechanisms of PU development. Advancements in proteomics technology allow the molecular determinants of complex samples like tissue (PUs) to be analyzed using MS.

## Materials and Methods

### Subject population and design

Tissue samples were collected from 23 subjects with SCI who were scheduled for PU surgery. For each patient, clinical data including age, sex, smoking status, stage of lesion (3 or 4), ASIA scale scores, and the presence of hypertension, atherosclerosis, dyslipidemia, and diabetes were collected. Exclusion criteria were PUs treated with negative pressure wound therapy, topical growth factors, or dressings containing proteins. The study was approved by the Ethics Committee of the Hospital Nacional de Parapléjicos (Toledo, Spain) and was conducted according to the principles of the Declaration of Helsinki. All patients signed written informed consent before inclusion.

Two different samples were collected from each patient, PU tissue and adjacent tissue as control. Samples were stored in phosphate-buffered saline (PBS) in sterile containers at 4°C and processed within a maximum of 2 h after surgery. Before analysis, samples were washed three times in PBS to reduce blood contaminants.

### Histological characterization of ulcer tissues

For histological characterization, one-third of each tissue was embedded in optimal cutting temperature compound (Thermo Fisher Scientific, Waltham, MA) and sectioned at 8 μm. Sections were stained with hematoxylin and eosin (H&E) to characterize the groups of study. Images were captured with an Olympus BX61 microscope connected to a DP71 color camera (Olympus, Tokyo, Japan).

### Proteomics pipeline

The experimental proteomics strategy consisted of two phases: (1) discovery phase using 12 tissue samples (6 control tissues and 6 PU tissues) analyzed by TMT followed by liquid chromatography tandem MS (LC-MS/MS). (2) Verification phase in an independent cohort of 8 tissue samples (8 controls and 8 PU tissues) by two different orthogonal techniques: western blotting and selected reaction monitoring (SRM). Three samples were used for WB optimization and for histological characterization.

### Sample preparation for proteomic analysis

For the discovery phase, tissue samples were cut into small pieces of ∼5 mm^2^ and placed inside centrifuge tubes containing ceramic beads (Roche, Penzberg, Germany) and 200 μL of lysis buffer (100 mM Tris pH 7.5, 1% sodium dodecyl sulfate [SDS] and 50 mM iodoacetamide [IAA]; Sigma-Aldrich, Madrid, Spain). Samples were then homogenized at 6,000 rpm/min for three cycles of 1 min using the MagNA Lyser Instrument (Roche Diagnostics GmbH, Mannheim, Germany). The lysate was then incubated for 5 min at 100°C, centrifuged at 18,000 *g* for 2 min, and the supernatant collected and incubated at room temperature for 10 min with shaking. Subsequently, samples were centrifuged at 18,000 *g* for 10 min and the supernatant was saved and assayed for protein concentration using the RC/DC Protein assay kit (Bio-Rad, Hercules, CA).

For the verification phase, tissue samples were ground into powder in liquid nitrogen with a mortar and 0.1–0.2 g was resuspended in 150–250 μL of protein extraction buffer (7 M urea, 2 M thiourea, 4% w/v CHAPS). The homogenate was sonicated in cycles of 2 min and then centrifuged at 18,000 *g* for 30 min at 4°C. The supernatant from this step was centrifuged at 14,000 *g* through a 0.22 μm pore size filter tube (Costar Spin-X Centrifuge Tube; Corning, Corning, NY) at 4°C to eliminate cellular debris and lipids. The protein concentration of the supernatant was determined by the Bradford-Lowry method (Bio-Rad Protein Assay).

### Protein digestion and isobaric labeling

We used a previously described protocol,^14^ with minor modifications. Quantitative differential LC-MS/MS analysis using TMT 10-plex isobaric labeling was developed with 100 μg of total protein, which was digested by the FASP protocol described previously,^15^ with minor adjustments. Samples were denatured by boiling for 5 min in 0.2% SDS, 50 mM IAA, and after incubating in the dark for 30 min at room temperature. Samples were then diluted in 7 M urea in 0.1 M Tris-HCl (pH 8.5; UA buffer) and loaded onto 10 kDa centrifugal filter devices (NanoSep 10k Omega; Pall Life Sciences, Ann Arbor, MI). Buffer was replaced by washing the filters with UA buffer and proteins were then reduced for 30 min with 10 mM Tris(2-carboxyethyl) phosphine hydrochloride (TCEP; Pierce, Rockford, IL), washed with 50 mM HEPES buffer, and alkylated for 20 min in the dark in 50 mM methyl methanethiosulfonate (MMTS; Pierce) in UA. Excess alkylating reagent was eliminated by washing three times with UA and further three times with 50 mM ammonium bicarbonate. Proteins were digested overnight at 37°C with modified trypsin (30:1 protein:trypsin [w/w] in 50 mM ammonium bicarbonate; Promega Corp., Madison, WI). The resulting peptides were twice eluted by centrifugation with 50 mM ammonium bicarbonate and 0.5 M sodium chloride. Trifluoroacetic acid (TFA) was added to a final concentration of 1% and the peptides were desalted onto C18 Oasis-HLB cartridges (Waters, Milford, MA) and dried-down for further analysis.

For stable isobaric labeling, the resulting tryptic peptides were dissolved in 100 mM triethylammonium bicarbonate (TEAB) buffer, and the peptide concentration was determined by measuring the amide bonds with the Direct Detect system (Millipore, Billerica, MA). Equal amounts of each peptide sample were labeled using 10-plex TMT Reagents (Thermo Fisher Scientific, Rockford, IL) previously reconstituted with 70 μL of acetonitrile (ACN) and, after incubation at room temperature for 2 h, the reaction was stopped by adding 0.5% TFA for 30 min. Samples were concentrated in a Speed Vac, desalted onto C18 Oasis-HLB cartridges and dried-down for further analysis. To increase proteome coverage, TMT-labeled samples were fractionated by high-pH reverse phase chromatography (High pH Reversed-Phase Peptide Fractionation Kit; Pierce) and concentrated as before.

### Protein identification and quantitation

Labeled peptides were analyzed by LC-MS/MS as previously reported.^14^ We used a C-18 reversed phase nano-column (75 μm I.D. × 50 cm, 2 μm particle size, Acclaim PepMap RSLC, 100 C18; Thermo Fisher Scientific, Waltham, MA) with a continuous ACN gradient consisting of 0–30% B in 360 min and 50–90% B in 3 min (A = 0.1% formic acid [FA]; B = 90% ACN, 0.1% FA). A flow rate of 200 nL/min was used to elute peptides from the nano-column to an emitter nanospray needle for real-time ionization and peptide fragmentation on an Orbitrap Fusion mass spectrometer (Thermo Fisher Scientific, Waltham, MA). An enhanced FT-resolution spectrum (resolution = 70,000) followed by the MS/MS spectra from the Nth most intense parent ions were analyzed along the chromatographic run. Dynamic exclusion was set at 40 s. For peptide identification, all spectra were analyzed with Proteome Discoverer (version 2.1.0.81; Thermo Fisher Scientific, Waltham, MA) using SEQUEST-HT (Thermo Fisher Scientific, Waltham, MA). For database searching at the UniProtKB/TrEMBL database containing all sequences from human and contaminants (May 14, 2016; 70,611 entries), the following parameters were selected: trypsin digestion with two maximum missed cleavage sites, precursor and fragment mass tolerances of 2 and 0.02 Da, respectively, TMT modifications at N-terminal and Lys residues as fixed modifications, and methionine oxidation, carbamidomethyl cysteine, and MMTS modified cysteine as dynamic modification. Peptide identification was performed using the probability ratio method,^16^ and the false discovery rate (FDR) was calculated using inverted databases and the refined method,^17^ with an additional filtering for precursor mass tolerance of 15 ppm.^18^ Identified peptides had an FDR ≤1%. Only those peptides were used to quantify the relative abundance of each protein from reporter ion intensities. Statistical analysis of quantitative data was performed using the weighted spectrum peptide and the protein (WSPP) statistical model previously described.^11^ In this model, the protein log2-ratios are expressed as standardized variables, that is, in units of standard deviation according to their estimated variances (Zq values).

### Western blotting

Protein samples obtained from PU and control tissues were resolved by 12% SDS–polyacrylamide gel electrophoresis using a Bio-Rad Miniprotean II electrophoresis cell run at a constant current of 25 mA/gel. After electrophoresis, the proteins were transferred to a nitrocellulose membrane under a constant voltage of 12 V for 60 min, which was then stained with Ponceau S. The membranes were then blocked for 1 h with PBS-T containing 2.5% nonfat dry milk and 5% bovine serum albumin (BSA). Membranes were incubated overnight with the primary antibody in PBS-T with 2% BSA. The primary antibodies used were rabbit polyclonal antisera against haptoglobin (HPT) (ab85846) (1/10,000) and alpha-1-antitrypsin (A1AT) (ab207303) (1/2,500) (both from Abcam, Cambridge, United Kingdom). After washing, the membranes were incubated with a specific horseradish peroxidase-conjugated secondary antibody in PBS-T containing 2% BSA. Detection was performed by enhanced chemiluminescence (ECL; GE Healthcare, Little Chalfont, United Kingdom). Densitometry was performed with ImageQuantTL software (GE Healthcare).

### Selected reaction monitoring

Following our previously published protocol,^19,20^ samples were reduced with 100 nM dithiothreitol (Sigma-Aldrich) in 50 mM ammonium bicarbonate (99% purity; Scharlau, Barcelona, Spain) for 30 min at 37°C and alkylated with 550 mM IAA in 50 mM ammonium bicarbonate for 20 min at room temperature. The proteins were digested in 50 mM ammonium bicarbonate, 15% ACN (LC-MS grade; Scharlau) with sequencing grade modified porcine trypsin (Promega Corp.) at a final concentration of 1:50. After digestion at 37°C overnight, 2% FA (99.5% purity; Sigma-Aldrich) was added and samples were cleaned with Pep-Clean spin columns (Pierce). Tryptic digests were dried in a Speed Vac and resuspended in 2% ACN, 2% FA before MS analysis. The LC-MS/MS system consisted of a TEMPO nano LC system (Applied Biosystems, Foster City, CA) combined with a nano LC autosampler and coupled to a modified triple quadrupole MS system (Applied Biosystems 4000 QTRAO LC/MS/MS). Three replicate injections (4 μL containing 20 μg of protein) were performed per sample using mobile phase A (2% ACN/98% water, 0.1% FA) with a flow rate of 10 μL/min for 5 min. Peptides were loaded onto a μ-Precolumn Cartridge (Acclaim Pep Map 100 C18, 5 μm, 100 Å; 300 μm I.D. × 5 mm; LC Packings, Idstein, Germany) to preconcentrate and desalt samples. Reverse phase LC was achieved on a C18 column (Onyx Monolithic C18, 150 × 0.1 mm I.D.; Phenomenex, Torrance, CA) in a gradient of phase A and phase B (98% ACN/2% water, 0.1% FA). Peptides were eluted at a flow rate of 900 nL/min in the following steps: 2–15% B for 2 min, 15–30% B for 18 min, 30–50% B for 5 min, 50–90% B for 2 min, and finally 90% B for 3 min. The column was then regenerated with 2% B for 15 additional minutes. Both the TEMPO nano LC and 4000 QTRAP system were controlled by Analyst Software v.1.4.5. The mass spectrometer was set to operate in positive ion mode with ion spray voltage of 2,800 V and a nanoflow interface heater temperature of 150°C. Source gas 1 and curtain gas were set to 20 and 20 psi, respectively, and nitrogen was applied as both curtain and collision gases. Collision energy was optimized to obtain maximum transmission efficiency and sensitivity for each SRM transition. A total of 94 SRM transitions (2–3 per peptide) were monitored during an individual sample analysis. They were acquired at unit resolution in both Q1 and Q3, with dwell times from 40 to 120 ms, resulting in cycle times of 4.0957 s. The IntelliQuan algorithm, included in Analyst 1.4.5 software, was used to calculate abundances based on peak areas after integration. Differentially expressed peptides were considered as those peptides with at least two of three transitions significant and, in the case of proteins identified by only one peptide, those with the same trend in both peptides.

### Functional group analysis

For functional examination of the identified proteins, a list of the 76 significantly varied proteins was entered into the online software Search Tool for the Retrieval of Interacting Genes/Proteins (STRING v9.1), for functional and protein interaction analyses.

### Statistical analysis

Statistical analyses were performed using SPSS 15.0 for Windows software (SPSS, Inc., Chicago, IL). Data of patient' characteristics are presented as mean in the case of continuous variables, or percentages in the case of discrete variables such as sex or the presence/absence of risk factors. For the TMT results, we considered proteins differentially expressed if they were identified with at least two peptides and they had log2-ratios expressed in the form of the standardized variables (Zq) ±1.5 (*p* ≤ 0.05), with Zq signifying the mean of the six replicates versus the internal standard. The changes in peptide and protein abundance were assessed with a 1% FDR, using the TMT reporter ion intensities from MS/MS scans from SanXoT software as inputs to the WSPP model.^21^ For SRM analysis, the Kolmogorov–Smirnov test was used to demonstrate normal distribution of data before use of the paired Student's *t*-test. Statistical significance was accepted at *p < 0.05, **p < 0.01, ***p < 0.001.

## Results

### Study population

During the course of the study, 23 patients were enrolled. The experimental proteomics strategy consisted of two phases: (1) discovery phase using 12 tissue samples (6 control tissues and 6 PU tissues) analyzed by TMT followed by LC-MS/MS. (2) Verification phase in an independent cohort of 8 tissue samples (8 controls and 8 PU tissues) by two different orthogonal techniques: western blotting and SRM. Three samples were used for WB optimization and for histological characterization. All patients presented severe PU (stage 3 or stage 4). Detailed clinical characteristics of the patients are given in Table 1.

**Table 1.** Clinical characteristics of patients recruited for the study

Patient	Age	Sex	PU Stage	ASIA Scale	AHT	CAHD	DM	Smoking	DL
1	34	Male	4	D4 ASIA A	No	No	No	No	No
2	42	Male	4	D7 ASIA A	No	No	No	No	No
3	50	Male	4	D8 ASIA D	Yes	No	No	Yes	No
4	24	Male	4	D7 ASIA A	No	No	No	Yes	No
5	76	Male	4	D4 ASIA A	Yes	Yes	No	No	Yes
6	22	Male	3	D11 ASIA A	No	No	No	No	No
7	48	Male	4	C4 ASIA B	No	No	No	No	No
8	35	Female	4	D5 ASIA A	No	No	Yes	Yes	Yes
9	61	Female	3	D4 ASIA A	No	No	No	Yes	No
10	28	Male	3	C5 ASIA A	No	No	No	No	Yes
11	37	Male	4	D3 ASIA A	No	No	No	No	Yes
12	48	Male	4	D2 ASIA B	No	No	Yes	No	Yes
13	41	Male	4	C6 ASIA A	No	No	No	No	Yes
14	70	Male	4	D10 ASIA A	Yes	No	No	No	Yes
15	35	Female	4	D5 ASIA A	No	No	No	Yes	No
16	72	Male	4	L1 ASIA A	Yes	No	No	No	No
17	66	Male	4	L1 ASIA A	No	No	No	No	No
18	47	Male	4	C7 ASIA A	No	No	No	Yes	No
19	29	Male	4	C6 ASIA A	No	No	No	No	No
20	53	Female	4	C5 ASIA A	No	No	No	No	No
21	67	Male	3	D4 ASIA A	Yes	No	No	No	Yes
22	56	Male	4	D3 ASIA A	Yes	No	Yes	No	Yes
23	39	Male	4	D6 ASIA A	Yes	No	Yes	Yes	No
Mean (%)	47	Male, 83	IV, 83	87% A, 8.7% B, 4.3% D	30%	4%	17%	30%	39%

AHT, arterial hypertension; ASIA, American Spinal Injury Association; CAHD, coronary artery heart disease; DM: type 2 diabetes mellitus; DL, dyslipidemia; PU, pressure ulcer.

### Histological changes of the severe pressure ulcers

**Figure 2.**
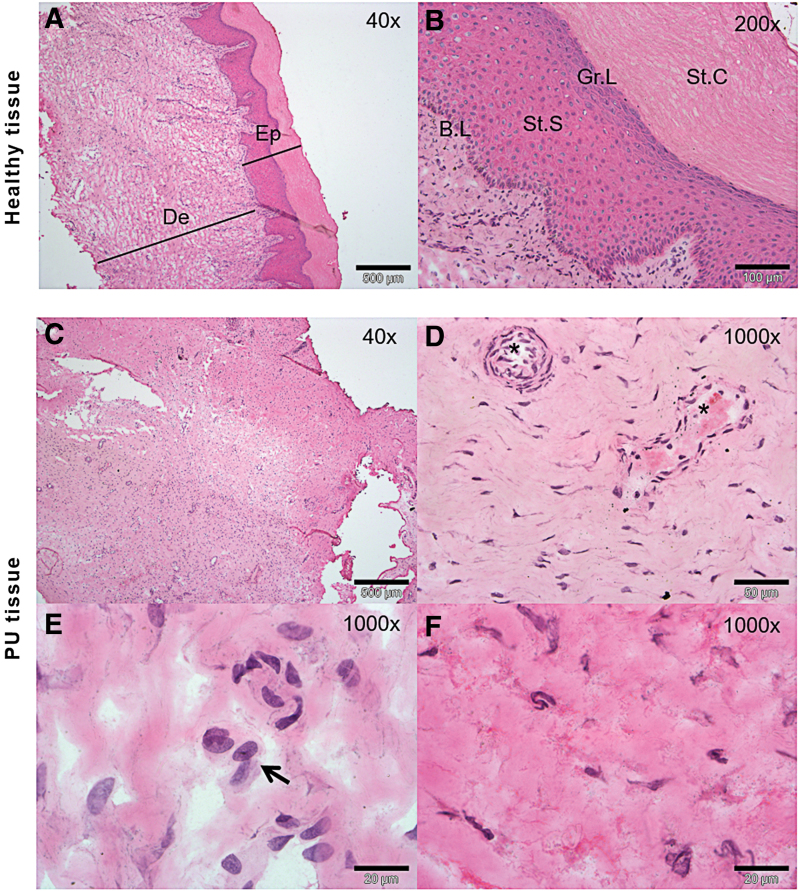
Histological characterization of grade 4 ulcer. **(A, D)** Control tissue showing the typical layer structure of the skin including epidermis (Ep) and dermis (De). In a larger magnification **(D,** 200 × **)**, stratum corneum (St.C), granular layer (Gr.L), stratum spinosum (St.S) and basal layer (B.L) are shown. **(B, C, E and F)** Tissue affected by severe PU showing loss of the epidermis layer (C), a less dense extracellular matrix **(E, F)**, numerous inflammatory cells **(***arrow*, **E),** and occluded blood vessels (*, **D)**. Scale bar corresponds to 500 μm in 40 × images, 100 μm in 200 × images and 20 μm in 1,000 × images. Color images are available online.

According to the histological examination by H&E staining, severe PU tissue samples showed possible inflammatory cells throughout the dermis, taking into account the existence of very important inflammation phase previous to a proliferation phase in PUs and, blood vessels were occluded or barely discernible (Fig. 2). In addition, the extracellular matrix was less dense and open dermal wounds were formed as a result of the pressure produced. By contrast, healthy tissue showed the typical layered structure, with well-packed collagen matrix and well-formed vasculature (Fig. 2).

### Proteomic profiling of PU tissue

The difference in abundance of protein between control and PU tissue was determined using a multiproteomic approach for identification of novel proteins and for further verification. In the discovery phase, samples from six patients (12 tissue samples) were analyzed using TMT-based multiplexed isobaric labeling followed by LC-MS/MS. This analysis allowed us to identify a total of 4,504 proteins, of which 76 showed abundance differences. Detailed information including protein ID, description of protein, Zq values, and *p*-values are given in Table 2.

**Table 2.** Differentially expressed proteins identified by tandem mass tag labeling in pressure ulcer tissue and control tissue

Uniprot ID	Name	Abbreviation	C	PU	p
Q9Y2V7	Conserved oligomeric Golgi complex subunit 6	COG6	−1.63	1.16	2.84E-05
P01008	Antithrombin-III	ANT3	−0.94	2.00	1.13E-04
P02652	Apolipoprotein A-II	APOA2	−1.56	2.72	3.91E-04
P02751	Fibronectin	FINC	−1.72	2.53	5.95E-04
P05155	Plasma protease C1 inhibitor	IC1	−1.23	1.71	6.88E-04
P02774	Vitamin D-binding protein	VTDB	−1.01	1.99	7.11E-04
P00751	Complement factor B	CFAB	−0.97	2.09	8.88E-04
P01023	Alpha-2-macroglobulin	A2MG	−1.35	2.16	1.15E-03
Q14624	Inter-alpha-trypsin inhibitor heavy chain H4	ITIH4	−0.78	1.86	1.28E-03
P29622	Kallistatin	KAIN	−1.04	1.54	1.50E-03
P02766	Transthyretin	TTHY	−0.74	1.74	1.57E-03
P01024	Complement C3	CO3	−0.98	2.12	1.76E-03
P04114	Apolipoprotein B-100	APOB	−1.04	2.33	1.84E-03
P00738	Haptoglobin	HPT	−1.39	2.42	1.85E-03
P02647	Apolipoprotein A-I	APOA1	−1.92	2.32	2.03E-03
P08603	Complement factor H	CFAH	−1.15	1.94	2.46E-03
Q14566	DNA replication licensing factor MCM6	MCM6	0.66	−2.29	2.64E-03
Q15166	Serum paraoxonase/lactonase 3	PON3	−0.52	1.72	2.69E-03
P02790	Hemopexin	HEMO	−1.02	1.68	2.71E-03
E9PGZ1	Caldesmon	E9PGZ1	−0.76	2.41	2.78E-03
Q9NR99	Matrix-remodeling-associated protein 5	MXRA5	−1.03	2.99	3.53E-03
Q14847	LIM and SH3 domain protein 1	LASP1	−0.54	1.77	3.58E-03
Q96D15	Reticulocalbin-3	RCN3	−1.11	1.84	4.05E-03
Q96AY3	Peptidyl-prolyl *cis*-*trans* isomerase FKBP10	FKB10	−0.64	2.56	4.20E-03
P78559	Microtubule-associated protein 1A	MAP1A	−1.56	0.51	4.45E-03
P01031	Complement C5	CO5	−1.21	1.60	4.50E-03
P01011	Alpha-1-antichymotrypsin	AACT	−1.23	1.98	4.65E-03
P01042	Kininogen-1	KNG1	−1.11	2.18	5.15E-03
P05546	Heparin cofactor 2	HEP2	−0.84	2.02	5.47E-03
P20908	Collagen alpha-1(V) chain	CO5A1	−0.96	2.21	5.48E-03
O15460	Prolyl 4-hydroxylase subunit alpha-2	P4HA2	−0.23	1.90	5.68E-03
Q8TD16	Protein bicaudal D homolog 2	BICD2	0.61	−2.15	5.69E-03
Q9BXN1	Asporin	ASPN	−2.79	2.28	6.14E-03
Q70UQ0	Inhibitor of nuclear factor kappa-B kinase-interacting protein	IKIP	−0.52	2.19	6.40E-03
Q86V35	Calcium-binding protein 7	CABP7	−0.33	2.17	7.95E-03
P23142	Fibulin-1	FBLN1	−1.69	1.90	8.01E-03
P07358	Complement component C8 beta chain	CO8B	−0.48	1.54	8.08E-03
Q99715	Collagen alpha-1(XII) chain	COCA1	−1.80	2.44	8.43E-03
P13674	Prolyl 4-hydroxylase subunit alpha-1	P4HA1	−0.50	1.94	8.45E-03
Q96IY4	Carboxypeptidase B2	CBPB2	−0.50	1.83	8.84E-03
P36955	Pigment epithelium-derived factor	PEDF	−1.23	1.93	8.86E-03
Q7LBR1	Charged multivesicular body protein 1b	CHM1B	−0.76	1.65	9.60E-03
P02787	Serotransferrin	TRFE	−1.08	1.89	1.01E-02
P02654	Apolipoprotein C-I	APOC1	−0.68	2.56	1.02E-02
Q9Y680	Peptidyl-prolyl *cis*-*trans* isomerase FKBP7	FKBP7	−0.65	2.21	1.15E-02
P35542	Serum amyloid A-4 protein	SAA4	−0.81	1.74	1.17E-02
P0C0L5	Complement C4-B	CO4B	−0.92	1.63	1.19E-02
P01009	Alpha-1-antitrypsin	A1AT	−1.40	2.09	1.26E-02
Q8N130	Sodium-dependent phosphate transport protein 2C	NPT2C	−0.72	2.11	1.26E-02
P01857	Immunoglobulin heavy constant gamma 1	IGHG1	−0.85	1.72	1.36E-02
P04003	C4b-binding protein alpha chain	C4BPA	−1.05	1.94	1.38E-02
P00450	Ceruloplasmin	CERU	−1.47	1.93	1.44E-02
Q14554	Protein disulfide-isomerase A5	PDIA5	−0.40	1.61	1.51E-02
Q14849	StAR-related lipid transfer protein 3	STAR3	−0.73	1.59	1.57E-02
P50454	Serpin H1	SERPH	−0.67	2.10	1.59E-02
Q9UEE9	Craniofacial development protein 1	CFDP1	−0.90	1.60	1.67E-02
Q15582	Transforming growth factor-beta-induced protein ig-h3	BGH3	−1.32	2.27	1.76E-02
Q15417	Calponin-3	CNN3	−0.58	1.91	1.99E-02
Q05682	Caldesmon	CALD1	−0.77	2.37	1.99E-02
P13611	Versican core protein	CSPG2	−1.57	0.84	2.07E-02
P0DOY2	Immunoglobulin lambda constant 2	IGLC2	−1.33	1.82	2.27E-02
P02656	Apolipoprotein C-III	APOC3	−1.59	1.98	2.29E-02
Q32P28	Prolyl 3-hydroxylase 1	P3H1	−0.48	1.68	2.46E-02
P01780	Immunoglobulin heavy variable 3–7	HV307	−1.19	2.03	2.78E-02
Q8TED1	Probable glutathione peroxidase 8	GPX8	−0.43	1.62	2.96E-02
P36222	Chitinase-3-like protein 1	CH3L1	−0.59	1.88	3.11E-02
Q5TCU3	Tropomyosin beta chain	Q5TCU3	−1.63	1.69	3.25E-02
O43488	Aflatoxin B1 aldehyde reductase member 2	ARK72	−1.57	0.50	3.47E-02
P14735	Insulin-degrading enzyme	IDE	0.84	−1.71	3.68E-02
Q15293	Reticulocalbin-1	RCN1	−0.94	1.86	3.74E-02
P02655	Apolipoprotein C-II	APOC2	−1.66	1.86	3.79E-02
P35442	Thrombospondin-2	TSP2	−1.74	1.69	4.44E-02
Q9UGM5	Fetuin-B	FETUB	−0.16	1.70	4.50E-02
Q9P2E9	Ribosome-binding protein 1	RRBP1	−0.50	1.59	4.70E-02
Q16352	Alpha-internexin	AINX	−0.73	1.79	4.88E-02
O75478	Transcriptional adapter 2-alpha	TAD2A	−0.31	2.72	4.96E-02

Zq values of controls (C) and ulcers (PU) and *p*-values are given.

**Figure 3.**
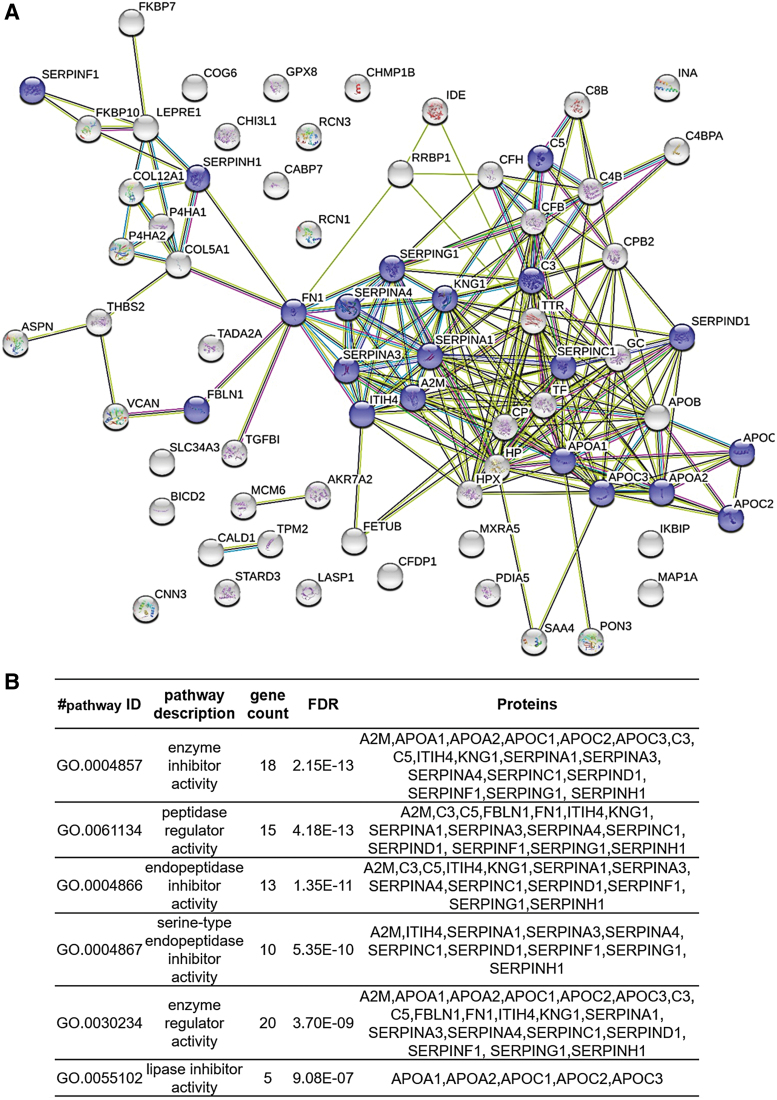
Pathway analysis of the differentially expressed proteins. **(A)** Protein–protein interaction networks were studied with STRING v9.1. Proteins with *red circles* correspond to proteins with enzyme regulator activity. **(B)** The six more significant molecular functions according to the classification performed using this tool are shown. Color images are available online.

Functional analysis of the proteins with differences in relative abundance was explored using STRING v10.5. According to the molecular function, it was remarkable that a substantial number of proteins were implicated in enzyme regulator activity, including peptidase regulator or lipase inhibitor activity categories (Fig. 3). This group included 20 proteins that were considered for further verification.

### A tissue protein pattern comprising 10 proteins reflects PU pathology

**Figure 4.**
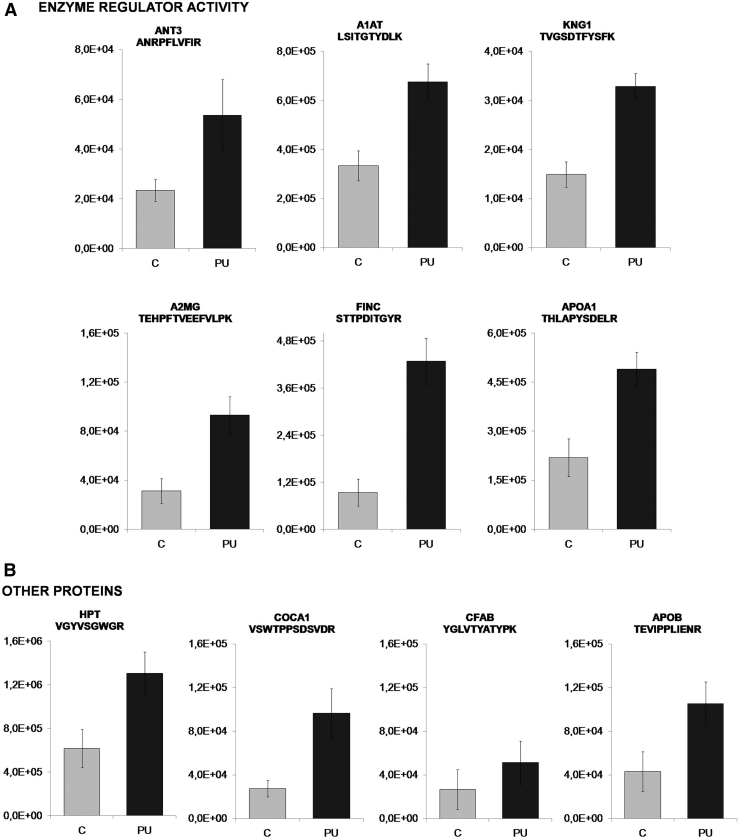
Verification of selected proteins showing increased abundance in PU than in control patients by SRM for **(A)** six enzyme regulator activity proteins, **(B)** other four proteins (*p*-value is shown).

**Figure 5.**
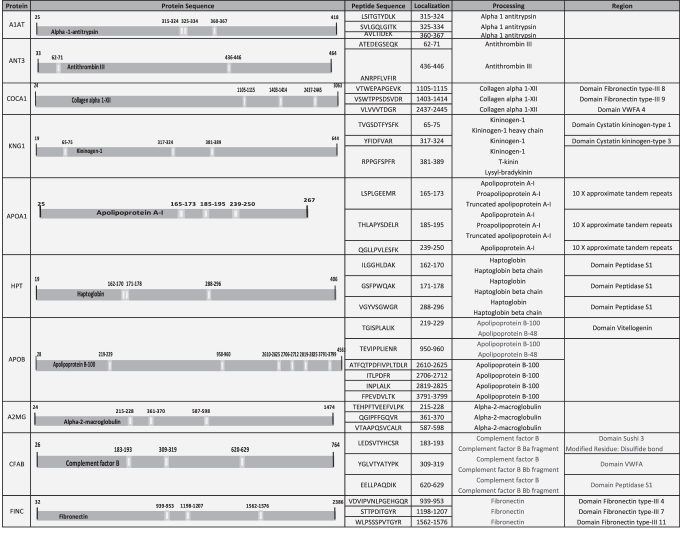
Localization of detected peptides in each protein used by SRM analysis. Table shows the position, in the complete amino acid sequence, of the detected peptides in each protein used for SRM analysis. All the detected peptides correspond to the mature protein. In addition, in some cases, some peptides correspond to some specific region, domain, or specific chain of the protein. ie) ILGGHLDAK, detected peptide in HPT, corresponds to mature protein (HPT), HPT beta chain, and domain peptidase S1. HPT, haptoglobin.

Our goal was to identify a protein pattern associated with PUs in SCI patients. According to functional analysis, we selected proteins with a role in enzyme regulator activity, including antithrombin-III (ANT3), A1AT, kininogen-1 (KNG1), alpha-2-macroglobulin (A2MG), fibronectin (FINC), and apolipoprotein A-I (APOA1). In addition, we selected four proteins with different yet potentially interesting functions in PU pathology: collagen alpha-1 (XII) chain (COCA1), HPT, apolipoprotein B-100 (APOB), and complement factor B (CFAB). All proteins were validated/confirmed using a complementary proteomics approach (SRM), and in an independent cohort of patients (*n* = 8) and control (*n* = 8) samples (Tables 3 and 4 and Fig. 4). Furthermore, we analyzed the location of the detected peptides in each protein used in SRM analysis. In Fig. 5, we showed that all detected peptides correspond to the mature protein.

**Table 3.** Results obtained from plasma analyses using selected reaction monitoring

Protein	Peptide Sequence	Fragment Ion	Mean Control-PU	p
A1AT	LSITGTYDLK	y6	−402,412.41 ± 249,809.7	1.30E-03
		y7	−437,057.95 ± 277,821.53	1.40E-03
		y8	−187,178.12 ± 126,128.55	2.00E-03
	SVLGQLGITK	y7	−509,022.33 ± 424,734.03	5.80E-03
		y8	−507,839.08 ± 418,632.1	5.40E-03
		y8	−585,621.83 ± 486,262.16	5.60E-03
	AVLTIDEK	y5	−115,106.04 ± 145,919.53	3.04E-02
		y6	−145,548.62 ± 175,934.17	2.59E-02
ANT3	ATEDEGSEQK	y3	−16,637.12 ± 20,649.96	2.83E-02
		y8	−47,715 ± 55,588.63	2.27E-02
		y8	−85,344.66 ± 99,636.77	2.29E-02
	ANRPFLVFIR	b5	−15,361.45 ± 22,748.96	4.88E-02
		y3	−54,335 ± 76,074.49	4.15E-02
		y4	−20,875.04 ± 30,447.39	4.68E-02
COCA1	VTWEPAPGEVK	b4	−15,812.47 ± 20,264.93	4.22E-02
		y5	−17,792.23 ± 22,085.95	3.85E-02
		y7	−97,762.04 ± 124,583.44	4.15E-02
	VSWTPPSDSVDR	b4	−27,023.12 ± 27,842.55	1.43E-02
		y8	−73,088.2 ± 76,156.36	1.50E-02
		y8	−107,295.37 ± 119,414.52	1.92E-02
	VLVVVTDGR	y5	−101,605.62 ± 118,758.53	2.30E-02
		y6	−121,889.75 ± 145,732.42	2.49E-02
		y7	−78,028.79 ± 88,883.14	2.10E-02
KNG1	TVGSDTFYSFK	y3	−7,413.29 ± 3,629.6	3.00E-04
		y5	−3,334.58 ± 2,316.47	2.30E-03
		y9	−43,284.66 ± 20,598.66	2.00E-04
	YFIDFVAR	b2	−52,029.87 ± 30,673.33	9.00E-04
		y5	−45,337.75 ± 27,993.15	1.20E-03
		y6	−41,239.45 ± 25,975.05	1.40E-03
	RPPGFSPFR	y2	−6,383.37 ± 5,545.46	6.90E-03
		y3	−18,495.2 ± 13,864.29	3.40E-03
APOA1	LSPLGEEMR	y5	−56,450.79 ± 43,014.42	3.70E-03
		y7	−16,943.95 ± 10,861.74	1.50E-03
		y7	−256,292.79 ± 190,775.88	3.30E-03
	THLAPYSDELR	b4	−281,834.16 ± 203,976.1	2.90E-03
		y4	−197,756.04 ± 131,621.54	1.80E-03
		y5	−330,919.2 ± 225,420.67	2.10E-03
	QGLLPVLESFK	y5	−34,867.87 ± 29,012.67	5.70E-03
		y7	−176,378.95 ± 137,634.88	4.20E-03
		y7	−136,489.2 ± 108,589.84	4.60E-03
HPT	ILGGHLDAK	y4	−41,925.29 ± 62,456.15	4.97E-02
		y7	−776,726.7 ± 1013,538.76	3.34E-02
		y8	−611,113.33 ± 810,442.31	3.51E-02
	GSFPWQAK	y5	−957,882.5 ± 888,115.59	9.20E-03
		y5	−385,635.54 ± 364,143.45	1.00E-02
		y6	−557,847.58 ± 514,062.62	9.00E-03
	VGYVSGWGR	b3	−583,046.87 ± 514,165.99	7.40E-03
		y5	−1098,829.29 ± 941,003.66	6.50E-03
		y6	−389,055.91 ± 348,505.66	7.90E-03
APOB	TGISPLALIK	b4	−7,465.45 ± 7,772.19	1.49E-02
		y6	−14,207.16 ± 16,923.08	2.46E-02
		y7	−12,995.37 ± 14,449.69	1.92E-02
	TEVIPPLIENR	b3	−89,423.91 ± 42,430.59	2.00E-04
		y7	−41,890.25 ± 17,452.57	1.00E-04
		y7	−55,614.25 ± 25,981.18	2.00E-04
	ATFQTPDFIVPLTDLR	b9	−1,192.77 ± 919.8	1.23E-02
		y6	−5,464.77 ± 3,043.13	3.50E-03
		y6	−6,478.88 ± 3,929.69	4.90E-03
	ITLPDFR	y2	−8,183.28 ± 5,275.17	3.10E-03
		y4	−18,439.52 ± 9,591.87	1.10E-03
		y5	−9,967.76 ± 4,647.03	6.00E-04
	INPLALK	y3	−3,000.87 ± 2,495.03	5.70E-03
		y5	−46,871.54 ± 26,318.56	7.00E-04
		y6	−5,041.95 ± 2,765.94	6.00E-04
	FPEVDVLTK	y6	−2,476.2 ± 1,219.94	3.00E-04
		y7	−2,911.37 ± 1,282.11	1.00E-04
		y8	−19,406.87 ± 9,560.03	3.00E-04
A2MG	TEHPFTVEEFVLPK	b6	−29,311.37 ± 22,724.35	4.00E-03
		b6	−83,588 ± 64,002.1	3.80E-03
		y3	−72,548.7 ± 51,048.4	2.50E-03
	QGIPFFGQVR	y5	−24,344.2 ± 21,201.5	7.00E-03
		y7	−89,972.41 ± 70,899.02	4.40E-03
		y7	−73,382.2 ± 58,061.97	4.50E-03
	VTAAPQSVCALR	b4	−74,965.16 ± 81,085.13	1.73E-02
		y8	−103,439.54 ± 110,066.7	1.62E-02
		y8	−111,140.54 ± 113,347.02	1.37E-02
CFAB	LEDSVTYHCSR	y10	−17,265.04 ± 18,653.53	1.72E-02
		y4	−4,135.41 ± 4,569.07	1.87E-02
		y9	−40,345.29 ± 42,142.31	1.51E-02
	YGLVTYATYPK	b3	−42,015.41 ± 32,497.17	4.00E-03
		y7	−19,895.83 ± 16,831.29	6.10E-03
		y8	−12,178.16 ± 8,061.39	1.80E-03
	EELLPAQDIK	b3	−2,826.08 ± 3,326.78	2.36E-02
		y6	−5,847.37 ± 8,655.82	4.88E-02
		y6	−1,150.79 ± 905.37	4.30E-03
FINC	VDVIPVNLPGEHGQR	b3	−282,587.2 ± 278,728.42	1.20E-02
		y11	−448,380.54 ± 441,255.04	1.19E-02
		y7	−53,274.08 ± 49,715.72	9.50E-03
	STTPDITGYR	y7	−291,683.45 ± 138,205.81	2.00E-04
		y7	−426,701.25 ± 206,751.66	3.00E-04
		y8	−286,076.79 ± 134,000.98	2.00E-04
	WLPSSSPVTGYR	b2	−119,508.25 ± 85,190.8	2.70E-03
		y10	−211,877.75 ± 146,830.83	2.30E-03
		y10	−375,549.87 ± 264,832.15	2.50E-03

The peptides and transitions measured for each protein and the statistical analyses for each transition, including mean and *p*-value. C, Control; PU, pressure ulcer.

**Table 4.** List of protein monitored by selected reaction monitoring including the experimental parameters

	Peptide Sequence	Precursor m/z	Precursor Charge	CE	DP	RT	Product m/z	Fragment Ion	Product Charge
A1AT	LSITGTYDLK	555.8057	2	27.40	71.60	21.69	696.3563	y6	1
							797.4040	y7	1
							910.4880	y8	1
	SVLGQLGITK	508.3109	2	24.70	68.20	24.39	716.4301	y7	1
							829.5142	y8	1
							415.2607	y8	2
	AVLTIDEK	444.7555	2	21.10	63.50	15.13	605.3141	y5	1
							718.3981	y6	1
ANT3	ATEDEGSEQK	547.2358	2	26.90	71.00	19.79	404.2140	y3	1
							921.3796	y8	1
							461.1934	y8	2
	ANRPFLVFIR	411.5803	3	19.80	61.10	27.50	586.3096	b5	1
							435.2714	y3	1
							534.3398	y4	1
COCA1	VTWEPAPGEVK	606.8166	2	30.30	75.40	17.97	516.2453	b4	1
							529.2980	y5	1
							697.3879	y7	1
	VSWTPPSDSVDR	673.3228	2	34.10	80.20	18.17	474.2347	b4	1
							872.4108	y8	1
							436.7091	y8	2
	VLVVVTDGR	479.2900	2	23.10	66.10	16.75	547.2835	y5	1
							646.3519	y6	1
							745.4203	y7	1
KNG1	TVGSDTFYSFK	626.2982	2	31.40	76.80	22.50	381.2132	y3	1
							691.3450	y5	1
							1,051.4731	y9	1
	YFIDFVAR	515.7715	2	25.10	68.70	28.85	311.1390	b2	1
							607.3198	y5	1
							720.4039	y6	1
	RPPGFSPFR	354.1944	3	18.10	56.90	17.70	322.1874	y2	1
							419.2401	y3	1
APOA1	LSPLGEEMR	516.2631	2	25.20	68.80	17.84	621.2661	y5	1
							831.4029	y7	1
							416.2051	y7	2
	THLAPYSDELR	434.5543	3	20.60	62.80	15.40	423.2350	b4	1
							532.2726	y4	1
							619.3046	y5	1
	QGLLPVLESFK	615.8583	2	30.80	76.00	34.59	623.3399	y5	1
							819.4611	y7	1
							410.2342	y7	2
HPT	ILGGHLDAK	308.5151	3	16.60	53.60	12.09	446.2609	y4	1
							349.1850	y7	2
							405.7271	y8	2
	GSFPWQAK	460.7349	2	22.00	64.70	21.15	629.3406	y5	1
							315.1739	y5	2
							388.7081	y6	2
	VGYVSGWGR	490.7511	2	23.70	66.90	18.31	320.1605	b3	1
							562.2732	y5	1
							661.3416	y6	1
APOB	TGISPLALIK	506.8237	2	24.60	68.10	28.98	359.1925	b4	1
							654.4549	y6	1
							741.4869	y7	1
	TEVIPPLIENR	640.8641	2	32.30	77.80	24.46	330.1660	b3	1
							838.4781	y7	1
							419.7427	y7	2
	ATFQTPDFIVPLTDLR	611.9964	3	26.10	75.70	37.36	511.2531	b9	2
							714.4145	y6	1
							357.7109	y6	2
	ITLPDFR	431.2451	2	20.30	62.60	24.66	322.1874	y2	1
							534.2671	y4	1
							647.3511	y5	1
	INPLALK	384.7525	2	17.70	59.20	19.12	331.2340	y3	1
							541.3708	y5	1
							655.4137	y6	1
	FPEVDVLTK	524.2897	2	25.60	69.30	24.19	674.4083	y6	1
							803.4509	y7	1
							450.7555	y8	2
A2MG	TEHPFTVEEFVLPK	558.2909	3	24.40	71.80	28.64	713.3253	b6	1
							357.1663	b6	2
							357.2496	y3	1
	QGIPFFGQVR	574.8142	2	28.50	73.00	28.98	606.3358	y5	1
							850.4570	y7	1
							425.7321	y7	2
	VTAAPQSVCALR	636.8401	2	32.00	77.50	15.27	343.1976	b4	1
							930.4826	y8	1
							465.7449	y8	2
CFAB	LEDSVTYHCSR	456.2067	3	21.20	64.40	8.92	627.2644	y10	2
							559.2405	y4	1
							562.7431	y9	2
	YGLVTYATYPK	638.3346	2	32.10	77.70	22.23	334.1761	b3	1
							843.4247	y7	1
							942.4931	y8	1
	EELLPAQDIK	578.3164	2	28.70	73.30	19.39	372.1765	b3	1
							671.3723	y6	1
							336.1898	y6	2
FINC	VDVIPVNLPGEHGQR	543.9618	3	23.90	70.80	22.56	314.1710	b3	1
							602.3151	y11	2
							780.3747	y7	1
	STTPDITGYR	555.7749	2	27.40	71.60	14.86	821.4152	y7	1
							411.2112	y7	2
							461.7351	y8	2
	WLPSSSPVTGYR	675.3461	2	34.20	80.30	21.69	300.1707	b2	1
							1,050.5215	y10	1
							525.7644	y10	2

CE, collision energy; DP, declustering potential; RT, retention time.

**Figure 6.**
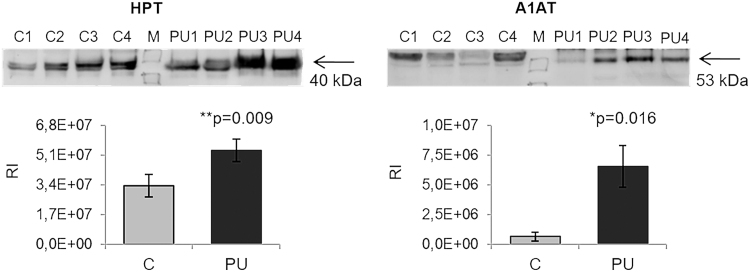
Verification of the differences observed with TMT labeling by western blotting. HPT and A1AT were analyzed by western blotting to confirm the reliability of SRM. * and ** showed statistical significance, *p < 0.05, **p < 0.01. A1AT, alpha-1-antitrypsin; RI, relative intensity.

To complement these findings, we performed western blotting of two representative proteins, HPT and CFAH, which were both found to be more abundant in PU tissue relative to control tissue (*p* = 0.009 and *p* = 0.016, respectively; Fig. 6).

## Discussion

The formation of PUs is an important and potentially life-threatening secondary complication of SCI, as they frequently lead to further functional disability and fatal infections, necessitating surgical intervention.^22,23^ The identification of new protein patterns involved in the pathogenesis of PUs may lead to better treatment management and also to new therapeutic interventions, which would help patients with SCI attain a better quality of life, facilitating their return to daily life. Although proteomics studies have been previously conducted on ulcers,^24–26^ to the best of our knowledge, this is the first proteomics study comparing healthy and PU tissue from patients with SCI, which is important given that the biochemical profile of chronic PU is different between SCI and non-SCI populations.^27^

Great efforts have been made in recent years to understand the mechanisms leading to the development of PUs in patients with SCI, including processes related to hemostasis, inflammation, proliferation, and remodeling.^28–30^ Evidence has shown that the pathological process of PU formation is characterized by increased levels of proinflammatory cytokines and proteases,^31^ and also reactive oxygen species,^32,33^ in addition to the development of a cellular senescent phenotype (keratinocytes, endothelial cells, fibroblasts, and macrophages).^34–37^ Moreover, there is a risk for persistent infection and a deficiency of stem cells, which are often also dysfunctional.^38–40^ The precise mechanisms of PU development, however, remain unclear. In this context, our findings may allow for a richer understanding of PU pathophysiology in SCI. Indeed, this is the first study to analyze differential protein abundance of PU tissue from SCI patients using high-throughput protein identification and quantification by TMT followed by LC-MS/MS.

We found a total of 76 proteins with differences in relative abundance between PU and control tissue using TMT labeling. Analysis of molecular function revealed a group of 20 proteins implicated in enzyme regulatory activity that were considered for further analysis. Finally, a group of 10 proteins (with 6 proteins related to enzymatic regulation) was confirmed by the complementary techniques of SRM and/or western blotting.

In relation to the group of six proteins with enzyme regulatory activity, five (A1AT, ANT3, KNG1, A2MG, and FINC) are associated with peptidase regulation, whereas APOA1 has lipase inhibitor activity. The study of the relationship between proteases and wound healing has mainly focused on metalloproteinases and their inhibitors.^41–43^ Of interest, in our study, we also found differences in other types of peptidase inhibitors, such as serine protease inhibitors (serpins) (Fig. 3).

We found more abundance levels of A1AT, a serpin that regulates the recruitment of neutrophils to sites of injury as a response to inflammation, thus acting as an anti-inflammatory and immunoregulatory protein.^44^ Similarly, ANT3 has powerful anti-inflammatory effects and modulates inflammatory responses by inhibiting thrombin and other factors, and by coagulation-independent effects, including direct interaction with cellular mediators of inflammation.^45–47^ The elevated abundance of these proteins could reflect a physiological but insufficient wound healing response. Consistent with this is the finding that an increase in ANT3 abundance favors the amelioration of other type of injuries such as burns.^48,49^ KNG1 has an antiprotease activity in the chronic wound environment^50^ and may undergo degradation, releasing specific proinflammatory peptides named kinins, which mediate vasodilatation, pain, and edema.^51–53^ Destruction of the extracellular matrix was clearly evident in the histological analysis of PU tissue, indicating an inhibition of fibrinolysis produced by inhibitors such as A2MG, which was also more abundant in injured tissue in our analysis. Increased levels of this protein have been reported in chronic ulcer wound fluid and correlate with FINC fragments produced by neutrophil elastases.^54^ FINC is a glycoprotein involved in many cellular mechanisms such as cell growth and migration,^55–59^ and like collagen, contains a number of binding sites for growth factors, including fibroblast growth factor, vascular endothelial growth factor, and platelet-derived growth factor, which have been shown to promote wound healing.^60–62^

With regard to lipase inhibitor activity, we found more abundance of APOA1 in PU compared with control tissue. APOA1 is the major structural component of high-density lipoproteins (HDL), and has anti-inflammatory and immunomodulatory functions in addition to endothelial protective properties.^63,64^ Of interest, a reduction in HDL levels are associated with increased foot ulceration in diabetic patients.^65^ HDL may beneficially impact wound healing by accelerating resolution of inflammation through enhancing granulation tissue formation, involving increased endothelial progenitor cell incorporation, and by accelerating reepithelialization.^66^

In the context of PU pathology, other proteins more abundant in PU tissue included HPT, COCA1, CFAB, and APOB. Similar to the role of the inhibitors mentioned previously, HPT is a natural inhibitor of collagen degradation,^67^ and its increased levels may reflect a compensatory mechanism to maintain collagen within normal limits, enhanced collagen degradation is related to tissue destruction or malfunction.^68,69^ Indeed, we found more abundance of COCA1, which may indicate an acceleration of cutaneous collagen synthesis for ulcer recovery.^70^ Components such as CFAB are involved in complement activation,^51,71,72^ and play an important role in inflammatory conditions.^28,73^ Finally, in contrast to APOA1, APOB is the primary component of low-density lipoproteins and may stimulate wound healing by inducing interleukin-8 secretion by fibroblasts.^74,75^

Overall, the results of our analysis suggest that damaged PU tissue is in the process of regeneration; however, these mechanisms are not sufficient to compensate for the pressure, friction, or shear forces that cause PUs. Typically, wound healing is defined by a complex interaction between proinflammatory cytokines, growth factors, proteases, and their inhibitors and extracellular components, which are in balance. In the setting of PUs, this balance is disrupted and the damage becomes chronic. In accordance, the proteins described in this study may have utility as new therapeutic options by supplementation, which may enhance the physiological response of wound healing.

### Study limitations

The major limitation of this study was the relatively small number of samples obtained, and as such it was necessary to include two types of PUs in the study, grades 3 and 4. Nonetheless, histological analysis indicated that both grades were largely similar, showing the absence of the epidermis layer as a result of the pressure produced in the lesion. To overcome the sample size limitation, we used two samples from ASIA B SCI patients in the verification phase, but the bulk of the work was performed with ASIA A SCI patients. Despite this limitation, patients were rigorously selected to be representative of elemental features such as age, sex, data of ASIA scale, advanced stage PU and metabolic control, in the case of diabetes. Finally, it is important to note that despite the reduced number of samples obtained, all experiments, in both discovery and verification phases were carried out with independent samples and not pools. Further studies are needed to prove the panel of biomarkers finding in this work and with higher sample size.

## Innovation

Great efforts have been made in recent years to understand the mechanisms leading to the development of PUs in patients with SCI. The identification of new protein patterns involved in the pathogenesis of PUs may lead to better treatment and new therapeutic interventions. In this innovative study, we analyzed, to the best of our knowledge, for the first time, differential protein abundance of PU tissue from SCI patients using high-throughput protein identification and quantification by TMT followed by LC-MS/MS. We found 76 proteins with difference in abundance between PU and control tissue using TMT labeling. The proteins described in this article may have utility as new therapeutic options by supplementation, which may enhance the physiological response of wound healing.

**Key Findings**

- This is the first proteomics study comparing healthy and PU tissue from patients with SCI.
- The innovative technique using high-throughput protein identification and quantification by TMT followed by LC-MS/MS allowed us to identify 76 proteins with difference in abundance between PU and control tissue.
- The proteins described in this article may have utility as new therapeutic options by supplementation, which may enhance the physiological response of wound healing.

## About the Authors

**Maria G. Barderas, PhD,** is the principal investigator (PI) at the Department of Vascular Physiopathology at the Hospital Nacional de Parapléjicos (HNP), SESCAM, Toledo (Spain) since 2006 and subsequently the Coordinator of the Proteomics Platform until 2013. **Laura Mourino-Alvarez, PhD**, is a postdoctoral researcher at the Department of Vascular Physiopathology at the HNP since 2015. **Montserrat Baldan-Martin, PhD,** is currently working at Hospital la Princesa. She performed her Doctoral thesis in the Department of Vascular Physiopathology at the HNP in 2017. **Tatiana Martin-Rojas, PhD,** works as postdoctoral researcher in the Department of Vascular Physiopathology at the HNP since 2016. **Nerea Corbacho-Alonso, MS,** is performing the Doctoral thesis since 2016 in the Department of Vascular Physiopathology at the HNP. **Juan Antonio Lopez, PhD,** is a group leader of Proteomics Unit at CNIC Carlos III (Madrid) for several years. **Tamara Sastre-Oliva, BS,** is lab manager in the Laboratory of Vascular Physiopathology at the HNP since 2013. **Felix Gil-Dones, PhD,** is Associate Professor in Europea University (Madrid) since 2014. **Jose Manuel Arevalo, MD,** is Specialist on Reconstructive and Aesthetic Plastic Surgery at the HNP.

## Abbreviations and Acronyms

A1AT: alpha-1-antitrypsin
A2MG: alpha-2-macroglobulin
ACN: acetonitrile
ANT3: antithrombin-III
APOA1: apolipoprotein A-I
APOB: apolipoprotein B
ASIA: American Spinal Injury Association
AUC: area under the curve
BSA: bovine serum albumin
CFAB: complement factor B
CE: collision energy
DTT: dithiothreitol
DP: declustering potential
FA: formic acid
FDR: false discovery rate
FINC: fibronectin
H&E: hematoxylin and eosin
HDL: high-density lipoproteins
HPT: haptoglobin
IAA: iodoacetamide
KNG1: kininogen-1
LC-MS/MS: liquid chromatography tandem mass spectrometry
MMTS: methyl methanethiosulfonate
PBS: phosphate-buffered saline
PUs: pressure ulcers
RI: relative intensity
RT: retention time
SCI: spinal cord injury
SDS: sodium dodecyl sulfate
SRM: selected reaction monitoring
STRING: search tool for the retrieval of interacting genes/proteins
TCEP: Tris(2-carboxyethyl) phosphine hydrochloride
TEAB: triethylammonium bicarbonate
TFA: trifluoroacetic acid
TMT: tandem mass tags
WSPP: weighted spectrum peptide and the protein
